# Improving Dietary Behavior Among Ethnic Minority Women in Denmark: A Feasibility Study Based on a Participatory and Culturally Adapted Intervention

**DOI:** 10.3390/ijerph16050795

**Published:** 2019-03-05

**Authors:** Anna Vera Jørring Pallesen, Stine Byberg, Maria Kristiansen

**Affiliations:** 1 Department of Public Health & Center for Healthy Aging, University of Copenhagen, 1014 Copenhagen, Denmark; makk@sund.ku.dk; 2 Steno Diabetes Center Copenhagen, 2820 Copenhagen, Denmark; stine.byberg@regionh.dk

**Keywords:** primary prevention, diet, behaviour change, ethnicity, social inequality

## Abstract

The Danish Heart Foundation and the non-governmental organization Neighborhood Mothers have co-developed a culturally adapted intervention seeking to promote healthy dietary behaviour among ethnic minority women. This feasibility study explores the potential of the intervention to reach ethnic minority women using health promotion initiatives. Participants attended instructor courses or cooking events, where culturally adapted, healthy recipes were introduced and meals prepared. Feasibility was explored using a mixed-method approach. Surveys were completed by 59 volunteers and 150 participants at five instructor courses and 21 cooking events. Individual interviews and focus group discussions were conducted with volunteers and participants after completion of the intervention. After the intervention, 61% of the 150 participants had high levels of knowledge about dietary recommendations, 96% intended to cook healthy dishes in the future and 84% intended to incorporate measuring equipment into their daily cooking routine. Participants with a high level of knowledge reported intention to change dietary behaviour more often than participants with lower levels of knowledge. Interviews confirmed that the participants cooked healthy dishes after participating, and incorporated knowledge about healthy food practices into their daily cooking. Few participants used measuring equipment. The intervention proved to be feasible as a health promotion initiative targeting a hard-to-reach population.

## 1. Introduction

Cardiovascular disease (CVD) is accountable for approximately 17.3 million annual deaths across the globe [1,2]. Social and ethnic inequalities exist in exposure to risk factors and in the burden of CVD. Studies find that an ethnic background is strongly associated with CVD incidence [3,4,5,6] with the risk being particularly high among non-Western migrants [3,4]. In Denmark, some groups of non-Western migrants have twice as high CVD incidence as ethnic Danes [3]. 

Disease prevention interventions play an important role in CVD prevention. Overcoming ethnic and socioeconomic inequalities in CVD requires disease prevention interventions that accommodate specific needs in terms of language, cultural and psychosocial factors in different population groups [7,8,9,10]. Diversity with regards to socioeconomic position, nationality, culture, language proficiency and, for migrants, time spent in the country of destination are important factors to consider in interventions [7,11]. This diversity, together with general barriers to disease prevention programs within ethnic minority groups, imposes a high demand for tailored disease prevention interventions. This in turn emphasizes the need to identify new and innovative ways to conduct disease prevention in people from varied backgrounds. Researchers within participatory research argue that a collaboration with the target population improves the long-term effects of interventions, as participants become an influential part of the decision-making and design process [12]. This influence and control increases the feeling of ownership among the participants, which has a positive effect on their engagement. Disease prevention interventions targeting people with an ethnic minority background should explore potential benefits of participatory approaches.

The Danish Heart Foundation, and Neighborhood Mothers, a Danish non-governmental organization, have collaboratively developed a culturally adapted intervention, seeking to promote healthy dietary behaviour among middle-aged and older ethnic minority women with the aim of preventing CVD. The core concept of the intervention is participatory approaches with a co-designed cookbook based on recipes chosen by the women and adjusted in collaboration with experts to ensure alignment with recommendations for healthy diets. The intervention is embedded within the social networks of female volunteers at Neighborhood Mothers with an ethnic minority background in order to reach ethnic minority women in their own communities. This study explores the feasibility of the intervention with regard to its potential as a preventive intervention targeting ethnic minority women. Specifically, we examine (1) its potential to motivate healthy dietary behaviour and (2) the cultural acceptability of the intervention. 

## 2. Materials and Methods 

### 2.1. The Intervention

The primary aim of the intervention was to increase awareness of healthy cooking practices and improve dietary behaviour among ethnic minority groups at high risk of CVD, through culturally acceptable education. The target group was ethnic minority women from non-Western countries living in communities characterised by low income. Women were targeted in this intervention for two reasons. Firstly, in these communities, families tend to perform traditional gender roles, where women primarily are in charge of choosing and cooking food for the family. Secondly, all volunteers were women, which made women from their local community an accessible target group.

The intervention consisted of two phases. During the first phase, volunteers at the Neighbourhood Mothers and dieticians from the Danish Heart Foundation (DHF) co-developed a cookbook with a variety of traditional recipes chosen by ethnic minority volunteers and later adapted to comply with the formal dietary recommendations advised by The Danish Veterinary and Food Administration [13]. Selected recipes from the cookbook can be viewed online [14]. This phase was based on participatory approaches, as ethnic minority women were highly involved in producing and selecting the recipes. In the second phase, DHF held five instructor courses in four areas with similar housing and socio-economic status in Denmark (two in Copenhagen and one in Helsingoer, Aarhus and Herning), inviting members of Neighbourhood Mothers with ethnic minority background to participate [termed ‘volunteers’]. Instructor courses focused on providing volunteers with insight and skills in terms of dietary recommendations and healthy cooking practices using the adapted versions of the traditional dishes from the cookbook. A core element in the education was the measurement of ingredients when cooking, as non-Western migrants tend to use more oil, butter and animal fat than recommended [15,16]. Implementing measuring equipment into their daily cooking was therefore perceived to be important in order to follow the recipes developed for healthy food practices. Furthermore, they were trained in communication skills and skills related to organising cooking events. After participating in the instructor course, the volunteers were encouraged to organise one cooking event each, inviting six to eight ethnic minority women from their community [termed ‘participants’] to join. In line with participatory approaches, the volunteers were responsible for all aspects of planning the cooking event and could organize it in any way they felt was best for the participants. A total of 47 events took place in different communities across the four cities. For most of the cooking events, the volunteers and participants shared the same ethnic background; however, some cooking events were held for participants with different ethnicities. During these events, the volunteers taught participants about dietary recommendations and healthy cooking practices using recipes from the cookbook. At these cooking events, participants received a set of recipes and measuring equipment to take home.

### 2.2. Study Design

This feasibility study was conducted using a mixed-method approach. After the training was completed, surveys were conducted. The survey for the volunteers consisted of 26 items and that for the participants had 30 items. Both surveys included items on demographic and socioeconomic information, level of knowledge regarding dietary recommendations, intention to change dietary behaviour, and satisfaction with the intervention. Items on demographic and socioeconomic information were developed according to recommendations in existing literature and similar national surveys in Denmark [17,18,19,20]. The item measuring level of knowledge asked the respondent to: “Tick all ten healthy food principles.” and were given 15 possible answers. Items regarding intention to change dietary behaviour and satisfaction with the intervention were inspired by items used in prior research, however, developed to fit the specific intervention [21,22]. The items were formulated as follows: “Do you wish to cook the dishes again?” and “Do you think that you will use measuring jugs and spoons in your daily cooking in the future?” The surveys were completed by volunteers at all five instructor courses and by participants at 21 out of 47 cooking events. In order to overcome language and cultural differences, the surveys were translated into four different languages (Turkish, Somali, Urdu and Arabic), in addition to the Danish version. The surveys were pilot tested prior to and after translation, among six persons with different ethnic minority backgrounds. Additionally, semi-structured individual interviews and focus group discussions were conducted with volunteers (*n* = 7) and participants (*n* = 8) one to two months after participation in a cooking event. The semi-structured interview guides were developed according to survey responses, of which some needed more exploration including experiences related to participating in the intervention. Furthermore, the interview guides comprised of questions regarding the impact of the intervention, including potential dietary changes the intervention might have led to. Recruitment for interviews was done through snowballing in which volunteers where asked after their cooking event whether they were willing to participate in an interview. The participants were asked in the survey whether they wished to be interviewed. Also, volunteers reached out to participants at the cooking events to help recruit candidates for interviews. We selected volunteers and participants for interviews using a maximum variation strategy to ensure diversity in ethnicity and place of residence.

### 2.3. Analysis

The study objectives were assessed through statistical analyses of the survey data and thematic analysis of the fully transcribed interviews. Descriptive statistics were used to describe the study population. We calculated chi^2^-tests to examine the crude associations between level of knowledge and intention to change dietary behaviour. All statistical analyses were undertaken using SPSS Statistics version 25 (SPSS Inc., Chicago, IL, USA). In the following paragraphs, we will explain how we conducted analyses specific for each objective. Individual interviews and focus group discussions were fully transcribed and analysed through thematic network analysis. Firstly, we identified basic themes in the interview transcriptions. These basic themes were then grouped into organising themes related to the study objectives. The organising themes were all connected to a global theme equivalent to the primary aim of the intervention.

#### 2.3.1. Motivating Healthy Dietary Behaviour

In the survey, intention to change dietary behaviour was measured using two items: whether respondents intended to (1) cook healthy versions of traditional dishes from the cookbook and, (2) use measuring equipment in their daily cooking routines. Responses to these items were explored using descriptive statistics. Furthermore, we analysed whether level of knowledge was associated with intention to change dietary behaviour. We examined this association as this intervention sought to motivate healthy dietary behaviour through improved knowledge on healthy food choices and cooking practices. Level of knowledge was determined taking information from one item in the survey that asked respondents to identify 10 dietary recommendations out of 15 possibilities. We chose to dichotomise the variable into a binary variable according to the distribution of responses. Thereby, high level of dietary knowledge was characterised by identifying 8–10 dietary recommendations, while low level of knowledge was characterised by identifying 0–7 recommendations. When examining the association between level of knowledge and intention to change dietary behaviour, we worked with the hypothesis that participants who had a high level of knowledge were more likely to express intention to improve dietary behaviour than participants with a low level of dietary knowledge. This hypothesis was formulated in line with existing literature, which indicates that knowledge increases the likelihood of changing dietary behaviour [23,24,25,26]. 

Whether the intervention motivated healthy dietary behaviour was moreover examined in semi-structured interviews in order to gain insights into whether the participants did in fact change their dietary behaviour after participating in the intervention

#### 2.3.2. Cultural Adaption

We examined cultural adaption in semi-structured interviews to investigate the importance of this aspect of the intervention, including the acceptance of the recipes in different cultural contexts and appropriateness of healthy cooking practices.

### 2.4. Ethical Procedures

This study was approved by the Danish Data Protection Agency (file number: SUND-2017-14). Furthermore, written informed consent was obtained from each interviewee and survey respondent.

## 3. Results

**Figure 1:**
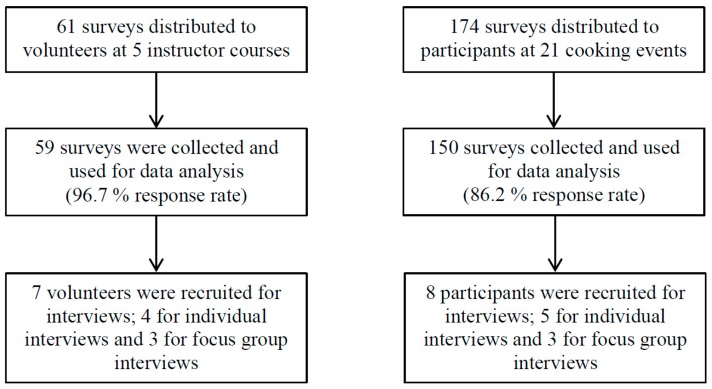
Data collection.

As presented in Figure 1, a total of five instructor courses were held with 59 out of 61 volunteers responding to the survey (97% response rate). Moreover, 47 cooking events were held with a total of 422 women participating. Data were collected at 21 cooking events with 150 out of 174 participants responding to the survey (82% response rate). We conducted individual interviews with four volunteers and five participants while focus group discussions were conducted with three volunteers and three participants. The interviews ranged from 11 min to 85 min (average: 56 min). An overview of the study population, showing the baseline characteristics including socioeconomic and demographic factors, is shown in Table 1.

### 3.1. Motivating Healthy Dietary Behaviour

As presented in Table 2, the vast majority of the participants (96%) reported an intention to cook the healthy versions of traditional dishes after participating in the intervention. Additionally, 84% of the participants intended to use measuring equipment, such as measuring jugs and spoons, in their daily cooking routines. In terms of level of knowledge, nearly 5% of the participants were not able to identify any of the dietary recommendations, while more than 25% were able to identify all. In more detail, 61% were able to identify 8–10 dietary recommendations—indicating a high level of knowledge; whereas 39% of the participants identified 0–7 recommendations—indicating limited knowledge about healthy food.

Table 3 presents the results from chi2-tests examining the crude associations between level of knowledge and the intention to change dietary behaviour. Of the participants who identified 8–10 healthy dietary recommendations, 87% reported the intention to use measuring equipment in their daily cooking routine, and 80% of the participants who identified 0–7 dietary recommendation reported the same intention. With regards to cooking the healthy versions of the traditional dishes, 98% of the participants who identified 8–10 healthy dietary recommendations reported the intention of doing so, while the same was the case for 93% of the participants who identified 0–7 dietary recommendations. The difference is small, and thus, the results only provide indications that participants who were able to identify 8–10 dietary recommendations were more likely to intend both to use measuring equipment and to follow the healthy recipes for the traditional ethnic dishes, compared to the participants who identified fewer dietary recommendations. Likewise, the association was statistically non-significant.

The interviews indicated that the intervention continued to affect the knowledge and food practices among volunteers and participants after completion of the intervention. Several of the volunteers and participants interviewed perceived that they were more conscious of their dietary behaviour and had begun to incorporate the dietary recommendations into their daily cooking routines. Measuring equipment, however, was not perceived to be relevant in the daily cooking routine, partly due to lack of time but also since these measuring practices were considered too onerous as it was much more natural for the participants to estimate ingredients by eye.

### 3.2. Cultural Adaption

In the interviews, the volunteers had a positive attitude towards the intervention’s foundation in using healthy versions of traditional dishes as reflected in the cookbook. Several volunteers described how they used the book actively when facilitating cooking events. For example, when choosing recipes according to the participants: if the participants were mainly from Pakistan, they would select two to three Pakistani recipes. Thus, the cooking events were culturally relevant for the participants. However, some volunteers experienced the participants having a negative attitude towards the recipes, as they contained less oil and more vegetables than the traditional versions. Nevertheless, in the end, the participants seemed to like the dishes: 

“I don’t know how many times she said that she was unhappy with it all [the food]... And the funniest thing was, when we were done, she thought it tasted good.”

It seemed to be challenging to implement the practice of measuring ingredients, especially of oil, at some of the cooking events. Family traditions and food culture influenced the attitudes of some of the participants towards the measuring equipment. A volunteer described one particular incident:

“There were some things that the participants just continued to be resistant towards… Then one of them said: ‘She put too little oil in [the dish]’ and they only needed two tablespoons of oil according to the recipe. Then the other one said: ‘If your mother-in-law sees that, she will be really mad.’”

Participants generally expressed satisfaction with the healthy versions of the traditional dishes, and the majority reported that they had introduced the dishes to their families and friends as described in the following quote: 

“We were supposed to make samosas together, me and my cousin, and then I told her: ‘I don’t think I want to make them with you.’ Because she wanted to deep-fry them and I didn’t want to do that. I said: ‘You should come home to me before Ramadan and taste it [an oven-baked samosa]. It tastes exactly the same. It tastes better in fact. It is... it is a bit more crispy.”

In terms of access and acceptability of the intervention, participants expressed that language barriers were overcome as the cooking events were carried out in the primary language of the participants. Furthermore, they perceived the cooking events to be accessible as the community-based approach made it easier to attend events that took place in familiar circumstances and networks.

## 4. Discussion

The findings of this study indicate that a culturally adapted CVD prevention intervention is feasible for reaching and engaging ethnic minority women of lower socioeconomic backgrounds. Following the intervention, the majority of the participants had a high level of knowledge about dietary recommendations, intended to cook healthy dishes in the future, and intended to incorporate measuring equipment into their daily cooking routine. Participants with high level of knowledge tended to report intention to change dietary behaviour to a higher extent than participants with a lower level of knowledge. The interviews confirmed that participants incorporated the knowledge about healthy food practices into their daily cooking, although measuring equipment was not perceived to be useful in their daily cooking.

### 4.1. Recruitment

Ethnic minorities, in particular non-Western groups and those with lower socioeconomic position are often harder to reach in disease prevention interventions due to barriers related to culture, social factors, language proficiency, and differences in health and risk perceptions [7,11]. This intervention managed to recruit these traditionally hard-to-reach participants from various ethnic backgrounds and low socioeconomic position (detailed information on the ethnic background of the participants is provided in Table S1 in the Supplementary Material). Managing to reach this population is primarily a result of the intervention design building on co-design of both content and delivery with volunteers with ethnic minority backgrounds. This played an important role in both the recruitment process and execution of the intervention. Furthermore, the community-based design, with cooking events being delivered in the neighbourhood of the volunteers, was important for access to and acceptability of the intervention. Using volunteers who share characteristics with the target population as a recruitment strategy to reach ethnic minorities is relatively unexplored. The high participation rates in this study indicate that this recruitment strategy could be feasible for future disease prevention interventions targeting ethnic minorities.

### 4.2. Motivating Healthy Dietary Behaviour

Dietary behaviour is negatively impacted by lack of knowledge regarding healthy food, and absence of skills on how to implement dietary recommendations in daily life [23,24,25,26]. This intervention sought to motivate behaviour change by filling potential gaps in knowledge regarding both dietary recommendations and the skills with which to implement them in daily life. Although, we recognise that the overwhelming majority of participants reported an intention to change dietary behaviour, this could partly be a result of social desirability bias, and could also be explained by the so-called ‘intention-behaviour gap’, whereby an intention may not necessarily lead to actual behavioural change. However, interviews with participants confirmed that the intervention had led to more healthy food choices among the interviewees two months after the intervention. In order to determine whether the intention to change dietary behaviour resulted in actual long-term behavioural change, a long-term follow-up is necessary. The ‘intention-behaviour gap’ has been identified in previous research and is described as a black-box of underlying psychological processes through which intention is translated into actual behavioural change [27]. The progression from intention to action is influenced by both internal and external factors including self-efficacy, risk awareness, knowledge, and psychosocial and structural factors [24,26]. Furthermore, these factors might differ according to individual characteristics such as gender, socioeconomic status and ethnicity [26]. According to interviewed participants, barriers to changing their dietary behaviour included food preferences of their husbands and children as well as the stressful and timely structured everyday life. These factors create a gap between intention and behaviour, which form a challenge in disease prevention, when seeking to make sustainable changes in the target population.

This intervention targets ethnic minority women who migrated to Denmark. This population has been exposed to a complex combination of risk factors affecting their health-related behaviour: in their country of origin, during the migration process, and in Denmark [28]. A large body of literature has examined the acculturation process, which migrants experience upon arrival in their host country. This process is identified as a multidimensional and dynamic process by which the attitudes, values, beliefs and behaviours of one culture are changed over time [29,30]. The acculturation process is affected by a wide range of factors, including social and contextual factors present in the particular local community in which people live [30]. In Denmark, the majority of migrants live in social housing in areas, which have a high concentration of residents with low income and low educational level [23,31]. Allen et al. (2014) found that greater acculturation among migrants living in low-income housing was associated with poorer diets. These findings indicate that migrants did possess knowledge of healthy dietary practices, and due to accessibility and cultural factors in their country of origin, they had healthier eating habits prior to their arrival to the host country, which is in line with findings from other studies [32,33]. Health promotion interventions should therefore help migrants maintain their healthy dietary behavior throughout the acculturation process in order to reduce their high risk of CVDs, as found in existing literature [3,4]. Drawing on prior knowledge and dietary practices of migrants provides a potential to develop an intervention with a particular high impact. It is therefore valuable to include the knowledge of the target population when designing an intervention and will increase cultural relevance, acceptability, and engagement in the target population. In addition, the accessibility of healthy and affordable food in the neighbourhood influences dietary behaviour [23,30]. Moreover, time is generally a crucial structural barrier for behavioural change, regardless of ethnic background. These social and contextual factors influence the long-term feasibility of interventions aiming to motivate changes in dietary behaviour, as participants might return to their usual habits despite their intention to change. In fact, interviews with volunteers and participants confirm this gap in regards to the use of measuring equipment. The survey responses given right after participation shows an intention to use measuring equipment among the vast majority; however, several interviewees indicated not having used them since due to structural factors and usual cooking routines. This intervention did therefore not manage to close the gap entirely. However, the results do indicate that the intervention was successful in reaching ethnic minorities—an otherwise difficult task in health promotion—as well as motivating healthier food choices. This is a result of the participatory approaches used in this intervention. Particularly, the role of the volunteers acting as role models for the participants. The volunteers and participants share characteristics and ethnic backgrounds, which creates familiarity and trust that the participants might not experience with health providers in the Danish healthcare system. This bond between volunteer and participants is the main strength of the intervention.

### 4.3. Cultural Adaption

Ethnic minority people have lower participation rates in disease prevention interventions, and evidence on how to effectively stimulate health behaviour change in ethnic minority populations is limited [7,8,9,10]. According to Davidson et al. (2013), adapted interventions are more likely to succeed in improving health behaviour, including healthy dietary habits. Specifically, adapted interventions consider the characteristics of their target population and context, in order to increase effectiveness and ensure the sustainability of the intervention [7]. When targeting ethnic minorities, it is crucial to consider their cultural background when designing an intervention. This particular intervention used cultural diversity as a foundation for the entire intervention. First and foremost, this intervention introduced culturally adapted recipes in line with dietary recommendations as an incentive to improve dietary behaviour among participants with ethnic minority backgrounds. Participatory approaches to disease prevention has a long tradition in international research; however, in Denmark this is a relatively new strategy in disease prevention targeting people with ethnic minority background. In Denmark, most disease prevention interventions seeking to improve healthy eating focus on dishes popular among ethnic Danes, thus neglecting the food preferences of ethnic minority groups. The findings of this study show the potential of tailoring successful interventions to the target group. Increased knowledge and skills do not necessarily translate directly into behaviour change; however, they are contributing factors [23,24]. Regardless of the short follow-up period, both volunteers and participants revealed in the interviews that they had continued to use the recommended recipes after participating, as well as using the dietary advice to modify other culturally specific recipes. Hence, this intervention has identified a mode of prevention that was feasible in reaching and engaging ethnic minority women in improving dietary behaviours.

### 4.4. Strengths and Weaknesses

The primary strength of this study is the participatory approach in which community members are actively involved in the development and organisation of the activities. Furthermore, the intervention was successful in recruitment of ethnic minority women who are often hard to reach in disease prevention interventions. Additionally, the response rate in this study is high considering that ethnic minorities tend to have lower participation rates [7,10].

Limitations of this feasibility study include the lack of a baseline survey, which would have made it possible to examine changes in the outcome measures before and after participating in the instructor courses and cooking events. It is, therefore, not possible to draw firm conclusions as to the full effect of the intervention in this study. Furthermore, the follow-up interviews were conducted one to two months after the cooking events, making the follow-up period relatively short. Therefore, long-term impacts of the intervention on the everyday lives of the volunteers and participants could not be determined. 

Data were only collected from 21 cooking events out of the 47 that were held. Consequently, the study population is small, which is problematic in the statistical analysis. Additionally, the study population might not be representative of the target population, as it contains relatively few people from each of the ethnic minority groups. The recruitment of participants for interviews might be subject to selection bias, as volunteers at the cooking events helped identify potential interviewees. 

According to several of the volunteers and participants, the surveys were somewhat long, which could influence their responses. Furthermore, the respondents filled out the survey while at the instructor courses and food events, whereby the context might have affected the responses. Additionally, the respondents might wish to appear as positively as possible, inducing a social desirability bias to the responses. This bias is particularly relevant when looking at the reported intention to change dietary behaviour as participants were asked to evaluate a cooking events held by women from their community. It is possible that the participants felt a need to report high levels of intention to change in order for them not to disappoint the women who held the cooking events. It could be seen as an attempt to help each other appear more successful, which may distort the results. However, the high reported rate of intention cannot be explained entirely by social desirability as the participants might in fact intend to improve their dietary behaviour. Furthermore, responses to the surveys are self-reported, which might lead to information bias. Interviews conducted in Danish might be influenced by the language proficiency of the interviewees, as poor Danish proficiency will limit the interviewees in their responses.

## 5. Conclusions

In conclusion, the intervention proved to be feasible as a CVD prevention intervention in terms of reaching ethnic minority women and positively motivating healthy dietary behaviour. However, the intervention was not successful in motivating participants to use measuring equipment in their daily cooking. Culturally relevant and acceptable health information was important for the ability to engage the target group. Furthermore, drawing on the commitment and community access of volunteers emerged as important. Future studies should investigate the long-term effects of the intervention in order to determine its effect on changing health behaviours in the longer term. Moreover, interventions could increase long-term engagement of participants by arranging for the participants, their families and members of their communities to meet periodically and cook the recipes. Additionally, health interventions targeting migrants should draw on the health knowledge already possessed by migrants and use this as a foundation for the content of intervention. This will likely increase the feeling of authority and improve the impact of the intervention. The impact of such interventions could additionally increase if ethnic minority men were also targeted as food traditions is formed by the social context and all members of that specific context.

## Figures and Tables

**Table 1:** Population characteristics.

Variable	Volunteers (*n* = 59) Frequency (%)	Participants (*n* = 150) Frequency (%)
Age		
17–24 years	2 (3.4%)	9 (6.5%)
25–34 years	4 (6.9%)	14 (10.1%)
35–44 years	19 (32.8%)	41 (29.7%)
45–54 years	25 (43.1%)	47 (34.1%)
55–64 years	5 (8.6%)	18 (13.0%)
65–74 years	3 (5.2%)	8 (5.8%)
75–84 years	-	1 (0.7%)
Missing	1	12
Continental region		
Europe	5 (8.6%)	22 (15.0%)
North Africa	6 (10.3%)	15 (25.2%)
East Africa	10 (17.2%)	17 (11.6%)
Middle East	27 (46.6%)	70 (47.6%)
South Asia	10 (17.2%)	23 (15.6%)
Missing	1	3
Number of years in Denmark		
1–10 years	9 (16.7%)	22 (19.5%)
11–20 years	17 (31.5%)	39 (34.5%)
21+ years	28 (51.9%)	52 (46.0%)
Missing	5	37
Marital status		
Married/living with partner	33 (55.9%)	96 (65.8%)
Divorced/separated	15 (25.4%)	18 (12.3%)
Unmarried/single	9 (15.3%)	16 (11.0%)
Widow	2 (3.4%)	14 (9.6%)
Other	-	2 (1.4%)
Missing	0	4
Highest completed level of education		
One or more shorter courses	25 (47.2%)	40 (35.1%)
Vocational education/skilled work	5 (9.4%)	20 (17.5%)
Short higher education	11 (20.8)	17 (14.9%)
Middle higher education	5 (9.4%)	24 (21.1%)
Long higher education	3 (5.7%)	7 (6.1%)
Another education	4 (7.5%)	6 (5.3%)
Missing	6	36
Employment status		
Employed full-time	7 (11.9%)	15 (11.3%)
Employed part-time	12 (20.3%)	20 (15.0%)
Student	8 (13.6%)	28 (21.1%)
Retired	9 (15.3%)	29 (21.8%)
Unemployed	14 (23.7%)	22 (16.5%)
Other	9 (15.3%)	19 (14.3%)
Missing	0	17

**Table 2:** Distribution of questionnaire responses with regard to level of knowledge and intention to change dietary behaviour.

Variable	Participants (*n* = 150) Frequency (%)
Number of identified healthy food principles	
0	7 (4.9%)
1	8 (5.6%)
2	3 (2.1%)
3	5 (3.5%)
4	6 (4.2%)
5	12 (8.5%)
6	4 (2.8%)
7	10 (7.0%)
8	26 (18.3%)
9	25 (17.6%)
10	36 (25.4%)
Missing	8
Intention to cook healthy versions of traditional dishes in the future	
Yes	120 (96.0%)
No	5 (4.0%)
Missing	25
Intention to use measuring equipment in daily cooking routine	
Yes	107 (84.3%)
No	20 (15.7%)
Missing	23

**Table 3:** Crude association between level of knowledge and intention to change dietary behaviour.

Identify the Ten Healthy Food Principles.	Will You Use Measuring Equipment in Your Daily Cooking Routine?			Will You Cook Healthy Version of the Traditional Dishes in the Future?		
	Yes	No	Significance	Yes	No	Significance
Identified 8–10	72 (86.7%)	11 (13.3%)	χ2: 1.1 *p* = 0.289	77 (97.5%)	2 (2.5%)	χ2: 1.4 *p* = 0.237
Identified 0–7	35 (79.5%)	9 (20.5%)		40 (93.0%)	3 (7%)	

## Supplementary Materials

The following are available online at https://www.mdpi.com/1660-4601/16/5/795/s1, Table S1: Population characteristic according to country of birth.
