# Supplementary material for: Improving Dietary Behavior Among Ethnic Minority Women in Denmark: A Feasibility Study Based on a Participatory and Culturally Adapted Intervention
Source: Int J Environ Res Public Health. 2019 Mar 5;16(5):795. doi: 10.3390/ijerph16050795 (PMC6427310; doi:10.3390/ijerph16050795)
Supplement: Supplementary file 1 [file ijerph-16-00795-s001.pdf]

Table S1. Population characteristic according to country of birth

Variable	Volunteers (n=59) Frequency (%)	Participants (n=150) Frequency (%)
Country of birth		
Denmark	4 (6,8 %)	20 (13,6 %)
Egypt	-	1 (0,7 %)
India	-	1 (0,7 %)
Iraq	4 (6,8 %)	19 (12,9 %)
Iran	-	2 (1,4 %)
Israel	-	1 (0,7 %)
Jordan	1 (1,7 %)	1 (0,7 %)
Kurdistan	-	3 (2,0 %)
Lebanon	9 (15,3 %)	17 (11,6 %)
Malaysia	1 (1,7 %)	-
Morocco	5 (8,5 %)	13 (8,8 %)
Norway	-	1 (0,7 %)
Pakistan	8 (13,6 %)	20 (13,6 %)
Palestine	-	1 (0,7 %)
Portugal	1 (1,7 %)	-
Saudi Arabia	-	1 (0,7 %)
Somalia	10 (16,9 %)	17 (11,6 %)
Sri Lanka	1 (1,7 %)	9 (6,1 %)
Sverige	-	1 (0,7 %)
Syria	11 (18,6 %)	17 (11,6 %)
Tunisia	1 (1,7 %)	1 (0,7 %)
Turkey	2 (3,4 %)	1 (0,7 %)
Missing:	0	3
